# Reports, excess risk and spatial distribution of domestic violence against women, Brazil, 2015-2020

**DOI:** 10.1590/S2237-96222025v34e20240277.en

**Published:** 2025-07-11

**Authors:** Lays Silva de Azevedo, Natan Nascimento de Oliveira, Rosana Rosseto de Oliveira

**Affiliations:** 1 Universidade de São Paulo, Faculdade de Saúde Pública, São Paulo, SP, Brazil; 2 Universidade Estadual de Maringá, Programa de Pós-Graduação em Enfermagem, Maringá, PR, Brazil

**Keywords:** Domestic Violence, Violence Against Women, Health Information Systems, Epidemiology, Descriptive, Geographic Mapping, Violencia Doméstica, Violencia contra la Mujer, Sistemas de Información en Salud, Epidemiología Descriptiva, Mapeo Geográfico

## Abstract

**Objective:**

To analyze reports, excess risk, and spatial distribution of domestic violence against women in Brazil between 2015 and 2020.

**Methods:**

Descriptive cross-sectional study with data from the Notifiable Health Conditions Information System. We calculated violence reporting rates per 100,000 and excess risk by Brazilian states. The rates were presented in tables, according to variables of interest, and via choropleth maps.

**Results:**

We analyzed 495,820 reports. There was a positive percentage change (29.4%) in the rates in the total period and a negative change (-16.6%) between 2019 and 2020. The 12-14 years age group had the highest rates (110.39 in 2015 and 165.63 in 2020); White women, mixed race women, and women with 8 years of schooling or more had the highest proportions (40%, 41.1%, and 43.8%, respectively). The states of Acre, Mato Grosso do Sul, Paraná, Minas Gerais, Tocantins and Rio Grande do Sul recorded high rates and excess risk >1, that is, higher than the national average.

**Conclusion:**

Violence showed significant variation during the study period, with an overall increase in rates and a decrease in 2020. Younger women had higher rates; those of White and mixed skin color and with higher education had higher proportions. In addition, some states had rates above the national average. These findings highlight differences in the distribution of domestic violence, reinforcing the need for approaches targeted at certain groups and locations, in addition to in-depth analyses and coordinated action to address this complex problem.

## Resumen

**Objetivo:**

Analizar las notificaciones, el exceso de riesgo y la distribución espacial de la violencia doméstica contra la mujer en Brasil, entre 2015 y 2020.

**Métodos:**

Estudio descriptivo transversal con datos del Sistema de Información de Enfermedades de Notificación Obligatoria. Se calcularon las tasas de notificación de violencia por cada 100.000 y el exceso de riesgo por estado. Las tasas se presentaron en tablas, según variables de interés, y en mapas coropléticos.

**Resultados:**

Se analizaron 495.820 notificaciones. Hubo una variación porcentual positiva (29,4%) en las tasas en el período total y una variación negativa (-16,6%) entre 2019 y 2020. El grupo de edad de 12 a 14 años presentó las tasas más altas (110,39 en 2015 y 165,63 en 2020); las mujeres blancas y de raza mixta y aquellas con 8 años de escolaridad o más tuvieron las proporciones más altas (40%, 41,1% y 43,8%, respectivamente). Los estados de Acre, Mato Grosso do Sul, Paraná, Minas Gerais, Tocantins y Rio Grande do Sul registraron tasas elevadas y exceso de riesgo >1, es decir, superiores al promedio del país.

**Conclusión:**

La violencia mostró una variación significativa en el período estudiado, con un aumento general de las tasas y una disminución en el año 2020. Las mujeres más jóvenes presentaron tasas más altas; y las blancas, de raza mixta y aquellas con niveles más altos de educación tenían proporciones más altas. Además, algunos estados tienen tasas superiores al promedio nacional. Estos hallazgos resaltan diferencias en la distribución de la violencia doméstica, lo que refuerza la necesidad de enfoques dirigidos a grupos y lugares específicos, así como análisis en profundidad y acciones coordinadas para abordar este complejo problema.

**Ethical aspects**

This research used public domain anonymized databases.:

## Introduction

Violence against women is a violation of human rights and can cause physical, sexual, financial or psychological harm to victims (1). Domestic violence is committed by anyone who has a family or emotional relationship with the victim, regardless of the degree of kinship, whether blood relatives or not, or the environment in which they live, and usually occurs in the domestic environment (2-4). 

This is a complex phenomenon that requires specific investigations and in-depth analysis, as well as actions by different agencies to address it (5). It is associated with numerous factors, such as low schooling levels and low socioeconomic status (6), and varies according to the region in which the victim lives, with variations between the most affected race/skin colors and schooling levels, among others, depending on the particularities of each location and its population (7).

The most common form of violence against women is perpetrated by an intimate partner or family member of the male sex, with the global prevalence of women who have suffered physical and/or sexual violence reaching 27%, according to 2018 estimates (1). A cross-sectional study conducted in New Orleans, United States, in 2020, showed that 10% of participants reported intimate partner violence (8). According to the Pan American Health Organization, 25% of women aged 15 to 49 in the Americas have suffered this form of violence; Brazil had a slightly lower estimate (23%). The highest estimate was found for Bolivia, 42%, and Peru, 38% (9).

Despite all the strategies adopted to combat and prevent violence against women, such as surveillance (10) and the *Maria da Penha* Law (11), representative rates are found in Brazil (1). With regard to women aged 15 to 49 who have suffered intimate partner physical and/or sexual violence, Brazil ranks 15^th^ among the countries of the Americas (9). Regarding total reports of violence nationally held on the Notifiable Health Conditions Information System (*Sistema de Informação de Agravos de Notificação* - SINAN) for the year 2015, 67.1% related to women (12). In 2023, 258,941 attacks against women considered to be domestic violence were recorded, representing an increase of 9.8% compared to the previous year, the total number of victims of threats increased by 16.5%, while victims of psychological violence and stalking increased by more than 30%, according to the country’s public security agencies (13). A study carried out in the state of Espírito Santo found that prevalence of physical and psychological violence was 39.3% and 57.6%, respectively, highlighting the high numbers of victims (14).

Therefore, it is necessary to deepen knowledge about its epidemiological aspects, such as the profile of victims and geographic variations, with a view to developing promotion, prevention and support actions. In addition, studies are needed that provide a panoramic view of the problem in Brazil, mainly aiming to provide support for health planning. 

In this context, the objective of this research was to analyze reports, excess risk and spatial distribution of domestic violence against women in Brazil, from 2015 to 2020. 

## Methods

### Design

This is a descriptive cross-sectional study of reports of domestic violence against women in Brazil from 2015 to 2020. 

### Setting

The unit of analysis was Brazil, with its 27 federative units spread over five major regions: North, Northeast, Midwest, Southeast and South. It had an estimated population of 213,317,639 inhabitants in 2021 and a Gini index of 0.544 in 2022 (15).

### Participants

The inclusion criteria were age group and relationship with the perpetrator. Female victims were between 12 and 59 years old and their perpetrators had some intimate relationship of affection: blood relatives or not blood relatives, spouses or ex-spouses, boyfriends or ex-boyfriends, or other forms of intimate relationships (2,3). Children under 12 years old were excluded because they receive different protection and treatment, according to the Child and Adolescent Statute, which recognizes distinct characteristics in child violence, including its forms, motivations and approaches to reporting, as well as specific procedures for support and protection (16). The National Policy for Comprehensive Child Health Care, in effect since 2015, also presents a strategic axis for comprehensive care for child victims of violence (17). Adolescents (12 to 19 years old) were included because they face forms of domestic violence similar to adult women, and represent a significant portion of the reports. Elderly women were excluded based on protection under the Elderly Statute, identifying violence against this population as a specific phenomenon, with particular motivations and differentiated treatment (18).

### Variables

The variables were selected based on the Individual Interpersonal/Self-Inflicted Violence Reporting Form:

- Characteristics of the victim

- Age group (in years: 12-14; 15-19; 20-29; 30-39; 40-49; 50-59);

- Race/skin color (White; Black; mixed race; Asian/Indigenous);

- Schooling (<8 years; ≥8 years);

- Marital status (single [single/widow/separated]; married); and

- Pregnant (yes; no).

- Characteristics of the violence

- Repetitive violence (yes; no);

- Type of violence (physical; psychological; sexual; other types of violence – torture, trafficking, financial violence, negligence, child labor, legal intervention, others);

- Form of aggression (bodily force/beating; strangling; blunt object; sharp object; threat; other means – hot substance/object, poisoning/intoxication, firearm or other means); and

- Sexual violence (harassment; rape; other types – child pornography, sexual exploitation, others);

- Characteristics of the perpetrator and motivation

- Relationship with the victim (father; mother; stepfather; stepmother; spouse; ex-spouse; boyfriend; ex-boyfriend; son; brother; other family member);

- Sex of the probable perpetrator (male; female; both);

- Suspected use of alcohol by the perpetrator (yes; no);

- Referral (specialized service; other services); and

- Motivation for violence (sexism; generational conflict; other motivations – homophobia, racism, religion, xenophobia, homelessness, disability, other).

### Data sources and measurement

The data were obtained on March 12, 2022 from the SINAN system, available via the Information Technology Department of the Brazilian National Health System. Individual data were collected according to the victim’s place of residence.

The descriptive analysis included absolute and relative frequencies and rates, calculated by the ratio between the cases of violence and the population in the period studied, multiplied by 100,000. The population was considered to be the average of the Brazilian Institute of Geography and Statistics estimates of women for the study period and location, obtained from the population projections for Brazil and its states, by sex and simple age. Rates were not calculated for race/skin color, schooling, marital status and pregnancy, as projections for these characteristics are not available.

Percentage change in violence reporting rates in Brazil was obtained with a 95% confidence interval (95%CI). The percentage change coefficient was obtained by subtracting the last year from the first, then dividing the result by the value of the first year, multiplied by 100; while confidence intervals were calculated by multiplying standard errors and a critical t-value of 1.96.

### Bias control

Processing was performed excluding all data that did not meet the inclusion criteria. This step was carried out meticulously, analyzing each variable with filters to minimize missing data and field completion errors that could lead to inappropriate exclusion. Missing data were those for which the field was “unknown” or left blank. This n and sample percentage were described in the results, allowing visualization of their representativeness.

### Statistical methods

We estimated excess risk of occurrence of domestic violence against women in the Brazilian states. This is a comparison between the number of cases and the number expected based on a reference risk, which is the aggregation of all cases in the country. Excess risk is obtained by comparing rates to a national or regional standard. In order to arrive at the expected number, we calculated the ratio between the total sum of cases and the sum of the populations considered in the study (19). Therefore, the calculation used to estimate excess risk for each state was the ratio between the number of events observed and the expected number. Excess risk <1 represented a state with risk lower than the national average, and excess risk >1 represented a state with risk higher than the national average (19).

Excel was used for tabulation, R 4.2.0 software for processing and descriptive analyses, GeoDa 1.20.0.0 for calculating rates and excess risk, and QGIS 3.16.16 for creating maps.

## Results

**Figure 1:**
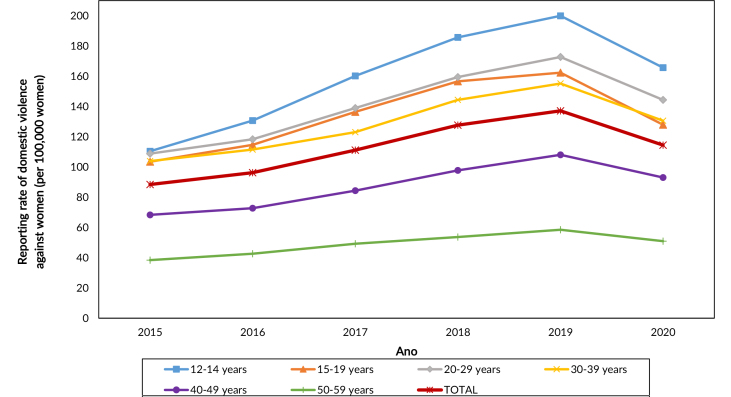
Rates of reported domestic violence against women (per 100,000), by age group. Brazil, 2015-2020 (n=495,820)

We analyzed 495,820 reports of domestic violence between 2015 and 2020 against women in the 12 to 59 age group in Brazil. Percentage change in rates from 2015 to 2020 was 29.4% (88.39 per 100,000 women in 2015; and 114.41 per 100,000 in 2020), with a negative change in 2020 compared to 2019: -16.6% (137.12 reports per 100,000 in 2019). In the same period, the highest rates of violence were in the 12 to 14 age group (110.39 cases per 100,000 in 2015; and 165.63 per 100,000 in 2020), followed by the 20 to 29 age group (108.81 per 100,000 in 2015; and 144.34 per 100,000 in 2020) (Figure 1).

White women accounted for 40% of reports, while mixed race women accounted for 41.1%, with 36,338 missing data (7.33%). In all regions of the country, the proportion of victims with higher schooling levels (>8 years) was higher, 43.8%. However, the number of reports for which the schooling variable field was “unknown” or left blank was high (29.5%). With regard to marital status, higher proportions of reports of single women were observed (47.2%), with 58,887 missing data (11.9%). In addition, 76.5% of the victims were not pregnant at the time of reporting, with 72,097 (14.5%) missing data (Table 1).

**Table 1:** Absolute numbers (n), proportions (%), reporting rates (Rate) and percentage change (% Change), and their 95% confidence intervals (95%CI), of domestic violence against women, by victim characteristics and regions of the country. Brazil, 2015-2020 (n=495,820)

Variables	North (n=34,219)		Northeast (n=69,367)		Southeast (n=266,601)		South (n=96,166)		Midwest (n=29,407)		Brazil (n=495,820)		Change (%)
	n (%)	Rate	n (%)	Rate	n (%)	Rate	n (%)	Rate	n (%)	Rate	n (%)	Rate	(95%CI)
**Age group** (years)													
12-14	7,525 (21.9)	1,498.9	8,037 (11.6)	579.0	14,274 (5.3)	827.0	9,749 (10.1)	1,679.8	3,588 (12.2)	1,013.8	43,181 (8.7)	948.9	50.04 (16.61; 83.47)
15-19	5,516 (16.1)	642.3	8,537 (12.3)	346.9	32,961 (12.4)	1,047.4	13,900 (14.4)	1,296.0	4,239 (14.4)	681.5	65,162 (13.1)	798.4	23.64 (-6.19; 53.47)
20-29	8,637 (25.2)	523.6	19,218 (27.1)	387.9	82,829 (31.1)	1,211.2	25,354 (26.4)	1,079.8	8,259 (28.1)	614.0	144,311 (29.1)	842.2	32.65 (5.80; 59.49)
30-39	7,504 (21.9)	510.7	18,808 (27.1)	397.7	74,715 (28.0)	1,031.1	23,601 (24.5)	1,013.1	7,305 (24.8)	531.9	131,952 (26.6)	769.5	25.67 (1.35; 49.98)
40-49	3,668 (10.7)	338.1	10,343 (14.9)	271.5	42,544 (15.9)	673.1	15,248 (15.9)	733.9	4,197 (14.8)	364.2	76,005 (15.3)	526.2	36.13 (10.53; 61.73)
50-59	1,369 (4.0)	188.3	4,424 (6.4)	149.7	19,278 (7.2)	351.5	8,314 (8.6)	433.6	1,819 (6.2)	208.0	35,209 (7.1)	294.4	32.55 (9.48; 55.63)
**Race/skin color**													
White	3,976 (11.6)	-	9,140 (13.2)	-	108,684 (40.8)	-	69,277 (72.0)	-	7,220 (24.5)	-	198,326 (40.0)	-	-
Black	2,339 (6.8)	-	7,555 (10.9)	-	30,854 (11.6)	-	6,106 (6.3)	-	2,476 (8.4)	-	49,332 (9.9)	-	-
Mixed race	25,442 (74.3)	-	45,957 (66.2)	-	100,995 (37.9)	-	15,974 (16.6)	-	15,199 (51.7)	-	203,589 (41.1)	-	-
Asian and Indigenous	1,673 (4.9)	-	1,062 (1.5)	-	2,613 (0.9)	-	999 (1.0)	-	1,885 (6.4)	-	8,235 (1.67)	-	-
**Schooling** (years)													
<8	12,734 (37.2)	-	21,224 (30.6)	-	59,402 (22.3)	-	30,111 (31.3)	-	8,923 (30.3)	-	132,409 (26.7)	-	-
≥8	15,411 (45.0)	-	25,671 (37.0)	-	119,666 (44.9)	-	45,787 (47.6)	-	10,609 (36.1)	-	217,167 (43.8)	-	-
**Marital status**													
Married	12,610 (36.8)	-	26,603 (38.3)	-	110,428 (41.4)	-	40,924 (42.6)	-	12,433 (42.3)	-	203,016 (40.9)	-	-
Single	18,564 (54.2)	-	34,347 (49.5)	-	120,301 (45.1)	-	47,752 (49.7)	-	12,917 (43.9)	-	233,917 (47.2)	-	-
**Pregnant**													
Yes	6,666 (19.5)	-	7,920 (11.4)	-	17,434 (6.5)	-	9,119 (9.5)	-	3,394 (11.5)	-	44,539 (8.9)	-	-
No	23,766 (69.4)	-	50,961 (73.5)	-	203,386 (76.3)	-	79,371 (82.5)	-	21,659 (73.6)	-	379,184 (76.5)	-	-

In all, 54.9% of the victims were suffering repetitive violence (80,766 uncategorized reports - 16.3%). Among the types of violence, physical violence had the highest rate (534.9 per 100,000), followed by psychological violence (295.2 per 100,000). The most frequent form of aggression was use of bodily force/beating (71.2%) and threats (28.2%). The sexual violence reporting rate was 70.7 per 100,000 (10.5%), with rape being the most evident type (51.1 per 100,000), whereby the Northern region stood out, with a sexual violence rate of 156.9 per 100,000 (Table 2).

**Table 2:** Absolute numbers (n), proportions (%) and reporting rates (Rate) of domestic violence against women, by violence characteristics and regions of the country. Brazil, 2015-2020 (n=495,820)

Variables	North (n=34,219)		Northeast (n=69,367)		Southeast (n=266,601)		South (n=96,166)		Midwest (n=29,407)		Brazil (n=495,820)	
	n (%)	Rate	n (%)	Rate	n (%)	Rate	n (%)	Rate	n (%)	Rate	n (%)	Rate
**Repetitive violence**												
Yes	20,690 (60.5)	328.9	38,955 (56.2)	191.9	140,984 (52.9)	458.3	56,396 (58.6)	546.2	15,395 (52.3)	269.1	272,447 (54.9)	371.2
No	9,100 (26.6)	144.6	18,490 (26.7)	91.1	78,054 (29.3)	253.7	28,413 (29.5)	275.2	8,524 (28.9)	149.0	142,607 (28.8)	194.3
Type of violencea												
Physical	20,053 (58.6)	318.7	50,759 (73.2)	250.1	230,279 (86.4)	748.6	68,409 (71.1)	662.5	23,053 (78.4)	402.9	392,602 (79.2)	534.9
Psychological	16,198 (47.3)	257.5	36,518 (52.6)	179.9	108,849 (40.8)	353.8	44,405 (46.2)	430.0	10,662 (36.3)	186.4	216,660 (43.7)	295.2
Sexual	9,875 (28.9)	156.9	11,713 (16.9)	57.7	16,554 (6.2)	53.8	9,544 (9.9)	92.4	4,172 (14.2)	72.9	51,869 (10.5)	70.7
Other types of violence	3,060 (8.9)	48.6	11,858 (17.1)	58.4	23,052 (8.6)	74.9	15,095 (15.7)	146.2	3,748 (12.7)	65.5	56,826 (11.5)	77.4
**Form of aggression** ^a^												
Bodily force / beating	17,119 (50.0)	272.1	41,926 (60.4)	206.6	210,461 (78.9)	684.2	63,313 (65.8)	613.1	20,067 (68.2)	350.8	352,927 (71.2)	480.8
Strangling	1,650 (4.8)	26.2	4,482 (6.5)	22.1	22,243 (8.3)	72.3	5,150 (5.4)	49.9	2,393 (8.1)	41.8	35,919 (7.2)	48.9
Blunt object	1,772 (5.2)	28.2	4,331 (6.2)	21.3	14,142 (5.3)	45.9	4,396 (4.6)	42.6	1,919 (6.5)	33.5	26,563 (5.4)	36.2
Sharp object	2,735 (7.9)	43.5	7,276 (10.5)	35.8	17,017 (6.4)	55.3	6,194 (6.4)	59.9	3,056 (10.4)	53.4	36,287 (7.3)	49.4
Threat	10,996 (32.1)	174.8	23,576 (33.4)	116.1	63,899 (23.9)	207.7	34,496 (35.9)	334.1	6,746 (22.9)	117.9	139,729 (28.2)	190.4
Other means	3,842 (11.5)	61.2	8,811 (13.4)	43.4	19,199 (7.5)	62.4	10,449 (10.9)	101.2	3,300 (11.6)	57.7	45,611 (9.2)	62.1
**Sexual violence**												
Harassment	2,272 (6.6)	36.1	2,682 (3.9)	13.2	4,928 (1.8)	16.0	2,740 (2.8)	26.5	869 (2.9)	15.2	13,492 (2.7)	18.4
Rape	7,040 (20.6)	111.9	8,249 (11.9)	40.6	11,749 (4.4)	38.2	7,156 (7.4)	69.3	3,281 (11.2)	57.3	37,484 (7.6)	51.1
Other types	920 (2.7)	14.6	1,492 (2.1)	7.3	1,606 (0.6)	5.2	868 (0.9)	8.4	391 (1.3)	6.8	5,279 (1.1)	7.2

^a^The victims may have suffered more than one type of violence and more than one type of aggression.

Violence rates were found to be the highest for spouses, followed by ex-spouses and boyfriends (291; 118.4 and 62.6 cases per 100,000, respectively). Regarding referrals of victims, when made, the highest rate was to non-specialized services, such as police stations, health networks, among others (673.5 per 100,000). The main motivation for violence was “other motivations”, however for 40% of the data the field was “unknown” or left blank (Table 3).

**Table 3:** Absolute numbers (n), proportions (%) and reporting rates (rate) of domestic violence against women, according to perpetrator characteristics, referral, motivation and region of the country. Brazil, 2015-2020 (n=495,820)

Variables	North (n=34,219)		Northeast (n=69,367)		Southeast (n=266,601)		South (n=96,166)		Midwest (n=29,407)		Brazil (n=495,820)	
	n (%)	Rate	n (%)	Rate	n (%)	Rate	n (%)	Rate	n (%)	Rate	n (%)	Rate
Perpetrator^a^												
Father	1,470 (4.3)	23.4	3,270 (4.7)	16.1	14,032 (5.3)	45.6	8,111 (8.4)	78.5	1,904 (6.5)	33.3	28,792 (5.8)	39.2
Mother	898 (2.6)	14.3	2,707 (3.9)	13.3	12,833 (4.8)	41.7	9,122 (9.5)	88.3	1,822 (6.2)	31.8	27,387 (5.5)	37.3
Stepfather	1,635 (4.8)	25.9	1,754 (2.5)	8.6	6,346 (2.4)	20.6	3,090 (3.2)	29.9	1,075 (3.7)	18.8	13,902 (2.8)	18.9
Stepmother	82 (0.2)	1.3	153 (0.2)	0.7	884 (0.3)	2.9	292 (0.3)	2.8	90 (0.3)	1.6	1,501 (0.3)	2.0
Spouse	12,748 (37.2)	202.6	29,557 (42.6)	145.6	117,386 (44.0)	381.6	40,475 (42.1)	391.9	13,400 (45.6)	234.2	213,589 (43.1)	291.0
Ex-spouse	6,754 (19.7)	107.4	13,062 (18.8)	64.3	46,128 (17.3)	149.9	16,601 (17.3)	160.8	4,338 (14.7)	75.8	86,892 (17.5)	118.4
Boyfriend	5,449 (15.9)	86.6	7,318 (10.5)	36.0	22,962 (8.6)	74.6	7,268 (7.6)	70.4	2,945 (10.0)	51.5	45,953 (9.3)	62.6
Ex-boyfriend	1,627 (4.7)	25.9	3,500 (5.0)	17.2	15,229 (5.7)	49.5	4,188 (4.3)	40.6	1,078 (3.7)	18.8	25,626 (5.2)	34.9
Son	526 (1.5)	8.4	1,725 (2.5)	8.5	10,625 (3.9)	34.5	3,839 (3.9)	37.2	1,110 (3.8)	19.4	17,825 (3.6)	24.3
Brother	1,463 (4.3)	23.2	3,546 (5.5)	17.5	16,665 (6.2)	54.2	4,303 (4.5)	41.7	1,487 (5.1)	25.9	27,467 (5.5)	37.4
Other family member	2,524 (7.4)	40.1	6,129 (8.8)	30.2	18,287 (6.9)	59.4	7,165 (7.4)	69.4	2,171 (7.4)	37.9	36,279 (7.3)	49.4
Perpetrator’s^a^ sex												
Male	31,745 (92.8)	504.6	62,427 (90.0)	307.6	230,833 (85.6)	750.4	80,976 (84.2)	784.2	25,467 (86.6)	445.2	431,500 (87.0)	587.9
Female	1,650 (4.8)	26.2	4,031 (5.8)	19.9	22,261 (8.3)	72.4	8,067 (8.4)	78.1	2,274 (7.7)	39.7	38,286 (7.7)	52.2
Both	536 (1.6)	8.5	1,728 (2.5)	8.5	9,364 (3.5)	30.4	6,648 (6.9)	64.4	1,337 (4.5)	23.4	19,616 (3.9)	26.7
**Suspected use of alcohol**												
Yes	11,840 (34.6)	188.2	26,140 (37.7)	128.8	96,755 (36.3)	314.5	36,007 (37.4)	348.7	13,211 (44.9)	230.9	183,975 (37.1)	250.7
No	15,849 (46.3)	251.9	27,041 (38.9)	133.2	103,293 (38.7)	335.8	38,869 (40.4)	376.4	9,539 (32.4)	166.7	194,622 (39.2)	265.2
Referral^b^												
Specialized service	14,154 (41.4)	224.9	27,862 (40.2)	137.3	85,962 (32.2)	279.4	32,647 (33.9)	316.2	8,955 (30.4)	156.5	169,602 (34.2)	231.1
Other services	29,359 (85.8)	466.7	55,386 (79.8)	272.9	256,837 (96.3)	834.9	121,866 (126.7)	1180.2	30,837 (104.9)	539.0	494,339 (99.7)	673.5
**Motivation for violence**												
Sexism	7,721 (22.6)	122.7	20,227 (29.2)	99.6	53,204 (19.9)	172.9	22,746 (23.6)	220.3	5,206 (17.7)	91.0	109,113 (22.0)	148.7
Generational conflict	2,909 (8.5)	46.2	5,574 (8.0)	27.5	37,239 (13.9)	121.0	9,206 (9.6)	89.2	2,663 (9.1)	46.5	57,599 (11.6)	78.5
Other motivations	7,630 (22.3)	121.3	15,868 (22.9)	78.2	67,002 (25.1)	217.8	32,002 (33.3)	309.9	8,094 (27.5)	141.5	130,617 (26.3)	177.9

^a^The victims may have more than one perpetrator; ^b^The victims may have been referred to more than one service.

**Figure 2:**
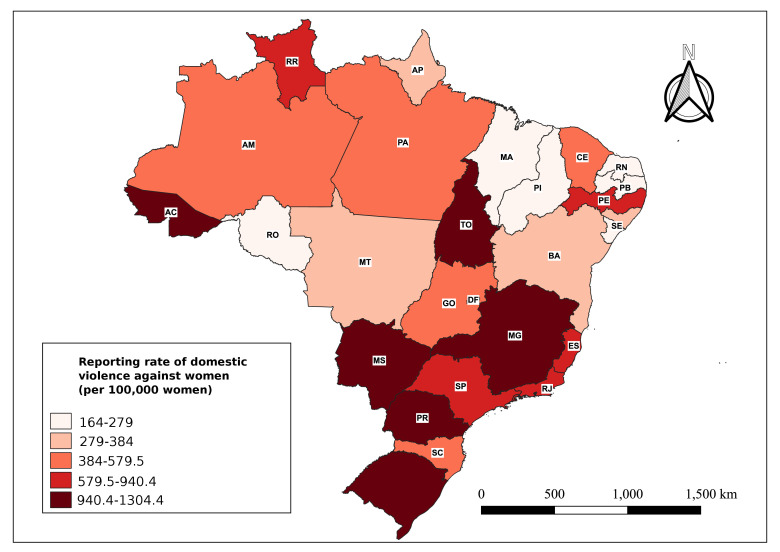
Reporting rates of domestic violence against women (per 100,000) in the federative units. Brazil, 2015-2020 (n=495,820)

As per the map showing distribution of violence reporting rates (Figure 2), the states with the highest values were Acre (1,304.45 per 100,000), Tocantins (971.71 per 100,000), Minas Gerais (1,020.53 per 100,000), Mato Grosso do Sul (1,271.74 per 100,000), Paraná (1,122.67 per 100,000) and Rio Grande do Sul (969.12 per 100,000). The states of Rondônia, Maranhão, Piauí, Rio Grande do Norte, Paraíba and Sergipe had the lowest rates in the country.

Regarding excess risk, the states where the risk of domestic violence against women was higher than the average for the entire country were: Acre (1.93), Mato Grosso do Sul (1.88), Paraná (1.66), Minas Gerais (1.51), Tocantins (1.43), Rio Grande do Sul (1.43), Rio de Janeiro (1.22), São Paulo (1.21) and Espírito Santo (1.17).

## Discussion

This study showed positive percentage change in the reporting rates of domestic violence against women in Brazil, over the period as a whole, and a decrease in 2020. The problem affects women of all races/skin colors and levels of schooling, and is widely distributed throughout Brazil, but the highest proportions can be seen among White, mixed race and better educated women, and some states have reporting rates above the national average.

The reduction in violence rates was evident in 2020, the year in which the COVID-19 pandemic began. Once social isolation was adopted to contain the spread of coronavirus (SARS-CoV-2), domestic violence against women worsened in several countries, since it occurs, in its vast majority, in situations of affection or cohabitation, and the social isolation measure made living together at home constant, which could result in increased arguments and tensions (20). In addition, fear of contamination and changes in women’s care services may also have caused a decrease in the number of women seeking to report violence to institutions (20). There was a 7.4% drop in reports of bodily injury due to domestic violence, but even so, 230,160 women filed reports (approximately 630 per day) (21); however, this does not necessarily imply a decrease in cases, since the changes imposed by quarantine increase the situation of vulnerability (22). In fact, data on telephone calls dialing 190 (the police emergency number) regarding domestic violence indicate a 16.3% increase in relation to 2019 (23), and records of femicide increased by 22.2% in March and April 2020 (21). The number of reports depends on numerous factors, such as the development of health services, or even the number of victims seeking care. According to the 2019 National Health Survey, 19.4% of women reported having suffered some type of violence; however, only 16.9% sought health care (24).

The highest reporting rates were found among younger women, aged 12 to 14 and 20 to 29, indicating the need for further research to understand causes and associated factors in these age groups. Records kept by public schools are relevant, constituting an important sector for referrals and reporting in school-age groups (25). In this study, data on adolescent victims were highly representative; in relation to this stage of life, schools can play an important role in reporting violence by identifying victims and referring them to appropriate services.

A study conducted with reporting data from 2008 to 2017 in the city of Rio de Janeiro showed that the age groups most affected by domestic violence were the 20-29 (29.1%) and 30-39 (22%) age groups (26), corroborating our findings, in which the highest percentages were seen in these age groups. A study conducted in Zimbabwe, with data from 2005, 2006, 2010, 2011 and 2015, identified that women aged 15 to 19 years are more likely to suffer domestic violence compared to older women, showing the vulnerability of this group to the problem, which may not understand the complexities of this violence within relationships (27).

There is also great proximity between the rates of violence reported against mixed race and White women. A study conducted in Brazil, with reporting data from 2009 to 2014, indicated that rates were higher for women of mixed race/skin color in the Northern, Northeast and Midwest regions, while rates for those of White race/skin color was more representative in the Southern and Southeast regions, largely corroborating our findings (7). However, in Brazil, self-reported mixed race people predominate, and it is not possible to state the proportions of the most affected victims (28).

We found higher rates of violence among women with higher levels of education. Although low levels of education are representative of studies with higher prevalence and as a risk factor, some studies have found higher rates of violence among victims with higher levels of education (26,29). Even so, women of all educational levels are affected (29).

The high rates of violence among women with higher levels of education may also be a reflection of access to information and the development of security, education and health institutions. Through advertising campaigns, these institutions disseminate relevant information about the problem, protective measures and women’s rights, thus encouraging women to become autonomous and break the cycle of violence (30).

It is also worth noting that most women were not experiencing domestic violence for the first time, characterizing repetitive violence, which has also been observed in other studies (26,31). A study carried out in 2020 in Salvador, Bahia, with women receiving legal aid due to conjugal violence, showed that aggressions suffered intensify over the course of the relationship, until they become more serious and less veiled (32). Persistence in the face of repetition often also constitutes a protection strategy, aiming to avoid more serious violence, such as femicide, where a woman’s resistance can have consequences if the perpetrator tries to prevent her from leaving him (33). External support is identified as one of the important factors for women to leave the situation of domestic violence in which they find themselves, along with the fear of the negative impacts of violence on physical and mental health and the need to protect their children (34).

Physical violence is the main type of violence found in this study, which is in line with the results reported in several studies (26,29,31). It has significant magnitude, especially due to the risk of causing obvious injuries (29). In our study, psychological violence also had high rates, diverging from the results of Silva & Oliveira (29) based on reporting data from 2009 to 2012 in the Federal District, where sexual violence appears immediately after physical violence, followed by psychological violence. However, it should be taken into account that, usually, psychological violence is one of the first manifestations, but is often ignored (32).

The most commonly used form of aggression was bodily force/beating, followed by threat, which is in line with a study conducted in the city of Rio de Janeiro, with reports from 2008 to 2017 (26), and contrary to the findings of another national study, for the period 2009-2014, in which all regions had a higher number of reports of threats (7).

Most victims were referred to non-specialized services. The service network for women in situations of violence has several entry points, which subsequently make referrals, but this is often not done appropriately, causing gaps in integration between services (35).

This study corroborates the fact that domestic violence transcends sociodemographic barriers, affecting all women. Therefore, the context in which the victims find themselves must be considered, and not just isolated factors (36,37).

In this study, the problem of domestic violence is distributed throughout the country. However, it was possible to identify the states with higher excess risk. States with high rates also presented excess risk >1.

The highest rates of violence were found in the states of Acre, Tocantins, Minas Gerais, Mato Grosso do Sul, Paraná and Rio Grande do Sul, similar to the findings of the spatial distribution carried out by another study (38). Yet another study indicated that the state of Mato Grosso do Sul had the highest rate of reports of violence against women (7), which is similar to the present study, since that state came in second place after Acre in terms of the highest rate and highest excess risk.

Use of secondary databases is one of the main limitations of our study, the variables of which present significant proportions of data filled in as “unknown”, “others”, “not applicable”, or left blank. However, the use of these records does not invalidate the findings, since all variables obtained completeness greater than 70%, except for the motivation variable, for which completeness was 59.97%, requiring caution in interpretation. Furthermore, we did not obtain the characteristics of violence considering all women in Brazil, due to the exclusion of children and elderly women. Notwithstanding, studies focused on these groups are necessary, since violence in these age groups has different treatment, other motivations and forms of aggression. To minimize data loss, processing was meticulous, with separate verification of each variable, aiming to minimize lack of information.

It was not possible to calculate rates by race/skin color, schooling, marital status or pregnancy, due to the lack of population estimates. Furthermore, the race/skin color data contained in the reports may be as perceived by the reporting health professional, also causing possible bias. In addition, and many health workers may not consider the importance of reporting events of violence and health service users may not recognize it as a responsibility of the health system, ignoring the need to report. These facts may contribute to the variation in reports based on the context of each of the country’s states.

However, domestic violence against women in Brazil showed significant variations between 2015 and 2020, with a continuous increase in rates in all years except in 2020, when there was a significant decrease. Younger women, especially between 12 and 14 years old, had higher reporting rates, while White, mixed race and better educated women had a higher proportion. In addition, the spatial analysis revealed states that had a higher rate of violence than the national average, resulting in greater excess risk. These findings highlight the inequalities in the distribution of domestic violence between population groups and regions of the country, reinforcing the need for targeted approaches, in addition to in-depth analyses and coordinated action by services and professionals to address it.

We therefore conclude that domestic violence against women is a complex and heterogeneous phenomenon, and this study is an important source of data, with relevant results on the scenario of the problem, contributing to the development of strategies to address it. In addition, it provides support for discussions, especially aimed at understanding the profile of victims, qualifying existing public policies and professional and institutional adaptation for effective care and combat.

## Data Availability

The database used for this study is available to the public at https://datasus.saude.gov.br/transferencia-de-arquivos/, using the following filters in Portuguese: fonte – SINAN, modalidade – dados, tipo de arquivo – viol (violência doméstica, sexual e/ou outras violências), ano – 2015 a 2020, UF – BR. The contents used for the study are also available. Below are the titles and respective URLs, accession numbers or DOIs of the files containing the contents underlying the text of the article: SINAN data: https:// datasus.saude.gov.br/wp-content/zipupload/Arq_162698079/arquivo.zip R Script for processing: https://github.com/laysaz/artigo1_disser_lays GQis project for mapping: https://github.com/laysaz/artigo1_disser_lays
