# Supplementary material for: Reports, excess risk and spatial distribution of domestic violence against women, Brazil, 2015-2020
Source: Epidemiol Serv Saude. 2025 Jul 11;34:e20240277. doi: 10.1590/S2237-96222025v34e20240277.en (PMC12266654; doi:10.1590/S2237-96222025v34e20240277.en)
Supplement: Translation

# Translation: Notificações, excesso de risco e distribuição espacial da violência doméstica contra mulheres, Brasil, 2015-2020

**DOI:** 10.1590/S2237-96222025v34e20240277.pt

**Authors:** Lays Silva de Azevedo, Natan Nascimento de Oliveira, Rosana Rosseto de Oliveira

**Affiliations:** 1 Universidade de São Paulo, Faculdade de Saúde Pública, São Paulo, SP, Brasil; 2 Universidade Estadual de Maringá, Programa de Pós-Graduação em Enfermagem, Maringá, PR, Brasil

**Keywords:** Violência Doméstica, Violência Contra a Mulher, Sistemas de Informação em Saúde, Epidemiologia Descritiva, Mapeamento Geográfico

## Abstract

**Objetivo:**

Analisar as notificações, o excesso de risco e a distribuição espacial da violência doméstica contra as mulheres no Brasil, entre 2015 e 2020.

**Métodos:**

Estudo transversal descritivo com dados do Sistema de Informação de Agravos de Notificação. Foram calculados taxas de notificação de violência por 100 mil e o excesso de risco por estado. As taxas foram apresentadas em tabelas, segundo variáveis de interesse, e em mapas coropléticos.

**Resultados:**

Foram analisadas 495.820 notificações. Houve variação percentual positiva (29,4%) nas taxas no período total e negativa (-16,6%) entre 2019 e 2020. A faixa etária de 12 a 14 anos obteve as maiores taxas (110,39 em 2015 e 165,63 em 2020); mulheres brancas, pardas e com escolaridade maior ou igual a 8 anos obtiveram as maiores proporções (40%, 41,1% e 43,8%, respectivamente). Acre, Mato Grosso do Sul, Paraná, Minas Gerais, Tocantins e Rio Grande do Sul registraram altas taxas e excesso de risco >1, ou seja, maior que a média do país.

**Conclusão:**

O agravo apresentou importante variação no período estudado, com aumento geral das taxas e queda em 2020. Mulheres mais jovens apresentaram maiores taxas; e brancas, pardas e com maior escolaridade apresentaram maiores proporções. Além disso, alguns estados possuem taxas acima da média nacional. Esses achados destacam diferenças na distribuição da violência doméstica, reforçando a necessidade de abordagens direcionadas a determinados grupos e locais, além de análises aprofundadas e ação coordenada para enfrentamento desse agravo complexo.

**Aspectos éticos**

Esta pesquisa utilizou bancos de dados de domínio público e anonimizados.:

## Introdução

A violência contra as mulheres é uma violação de direitos humanos, podendo ocasionar danos físicos, sexuais, patrimoniais ou psicológicos às vítimas (1). A violência doméstica é aquela cometida por qualquer pessoa que possua relação familiar ou afetiva com a vítima, independentemente do grau de parentesco, consanguíneo ou não, e do ambiente de convivência, ocorrendo normalmente no ambiente doméstico (2-4). 

Trata-se de um fenômeno complexo, que necessita de investigações e aprofundamentos específicos, bem como de ações de combate por diferentes órgãos (5). Está associada a inúmeros fatores, como baixa escolaridade e baixa condição socioeconômica (6), e possui diferenças conforme região de residência da vítima, podendo haver variações na raça/cor da pele e escolaridade mais atingida, entre outros, variando a partir das particularidades de cada localidade e sua população (7).

A forma mais comum de manifestação tem o parceiro íntimo ou familiar do sexo masculino como agressor, chegando a 27% a prevalência global de mulheres que já sofreram violência física e/ou sexual, conforme estimativas de 2018 (1). Uma pesquisa transversal realizada em New Orleans, Estados Unidos, em 2020, demonstrou que 10% das participantes relataram violência por parceiro íntimo (8). Segundo a Organização Pan-Americana da Saúde, 25% das mulheres de 15 a 49 da região das Américas já sofreram essa violência; o Brasil apresenta uma estimativa pouco abaixo (23%). A maior estimativa é observada na Bolívia, 42%, e no Peru, 38% (9).

Apesar de todas as estratégias adotadas para combate e prevenção da violência contra mulheres, como vigilância (10) e a Lei Maria da Penha (11), são observadas taxas representativas no Brasil (1). Quando observadas mulheres com idade de 15 a 49 anos que já sofreram violência física e/ou sexual por parceiro íntimo, o país ocupa a 15ª posição entre os países das Américas (9). No Sistema de Informação de Agravos de Notificação (Sinan), dentre o total de notificações de violência nacional em 2015, 67,1% eram de mulheres (12). Em 2023, foram registradas 258.941 agressões contra mulheres consideradas violência doméstica, apresentando um aumento de 9,8% em relação ao ano anterior, e o total de vítimas de ameaça subiu 16,5%, enquanto vítimas de violência psicológica e perseguição tiveram aumento de mais de 30%, segundo os órgãos de segurança pública do país (13). Um estudo realizado no Espírito Santo obteve prevalência de 39,3% e 57,6% de violência física e psicológica, respectivamente, ressaltando os altos números de vítimas (14). 

Portanto, é necessário aprofundar o conhecimento acerca de seus aspectos epidemiológicos, como o perfil das vítimas e variações geográficas, visando à elaboração de ações de promoção, prevenção e assistência. Além disso, fazem-se necessário estudos que possibilitem uma visão panorâmica do agravo no país, visando principalmente prover subsídios para o planejamento em saúde. 

Nesse contexto, o objetivo desta pesquisa é analisar as notificações, o excesso de risco e a distribuição espacial da violência doméstica contra as mulheres no Brasil, no período de 2015 a 2020. 

## Métodos

### Delineamento

Estudo transversal descritivo das notificações de violência doméstica contra mulheres referentes ao período de 2015 a 2020. 

### Contexto

A unidade de análise foi o Brasil, com suas 27 unidades federativas (UF) dispostas em cinco grandes regiões: Norte, Nordeste, Centro-Oeste, Sudeste e Sul. Possui população estimada de 213.317.639 pessoas em 2021 e índice de Gini de 0,544 em 2022 (15).

### Participantes

Os critérios de inclusão foram faixa etária e relação com agressor. As vítimas do sexo feminino tinham de 12 a 59 anos e seus agressores mantinham alguma relação íntima de afeto: familiares consanguíneos ou não, cônjuges ou ex-cônjuges, namorados ou ex-namorados, ou outra relação íntima (2,3). Crianças abaixo de 12 anos foram excluídas por terem proteção e tratativas diferenciadas, em função do Estatuto da Criança e do Adolescente, que reconhece características distintas na violência infantil, incluindo suas formas, motivações e abordagens para notificações, bem como procedimentos específicos de assistência e proteção (16). A Política Nacional de Atenção Integral à Saúde da Criança, de 2015, também apresenta um eixo estratégico para atenção integral às crianças vítimas de violência (17). Adolescentes (12 a 19 anos) foram incluídas, pois enfrentam formas de violência doméstica semelhantes às mulheres adultas, e representam parcela significativa das notificações. Idosas foram excluídas com base na proteção pelo Estatuto do Idoso, identificando-se a violência contra essa população como um fenômeno específico, com motivações particulares e tratativas diferenciadas (18). 

### Variáveis

As variáveis foram selecionadas com base na Ficha de Notificação Individual de Violência Interpessoal/Autoprovocada: 

**Características da vítima**

- Faixa etária (em anos: 12-14; 15-19; 20-29; 30-39; 40-49; 50-59);

- Raça/cor da pele (branca; preta; parda; amarela/indígena);

- Escolaridade (<8 anos; ≥8 anos);

- Situação conjugal (solteira [solteira/viúva/separada]; casada); e

- Gestante (sim; não).

**Características da violência**

- Violência de repetição (sim; não);

- Tipo de violência (física; psicológica; sexual; outros tipos de violência – tortura, tráfico, violência financeira, negligência, trabalho infantil, intervenção legal e outros);

- Meio de agressão (força corporal/espancamento; enforcamento; objeto contundente; objeto perfurocortante; ameaça; outros meios – substância/objeto quente, envenenamento/intoxicação, arma de fogo e outro meio); e

- Violência sexual (assédio; estupro; outros tipos – pornografia infantil, exploração sexual e outro);

**Características do agressor e motivação**

- Vínculo com a vítima (pai; mãe; padrasto; madrasta; cônjuge; ex-cônjuge; namorado; ex-namorado; filho; irmão; outro familiar);

- Sexo do provável autor (masculino; feminino; ambos);

- Suspeita de uso de álcool pelo autor (sim; não);

- Encaminhamento (serviço especializado; outros serviços); e

- Motivação da violência (sexismo; conflito geracional; outras motivações – homofobia, racismo, religião, xenofobia, situação de rua, deficiência e outras).

### Fontes de dados e mensuração

Os dados foram obtidos do Sinan, disponíveis no Departamento de Informática do Sistema Único de Saúde, em 12 de março de 2022. Foi realizada a coleta de dados individuais conforme o local de residência da vítima. 

A análise descritiva incluiu frequências absolutas, relativas e taxas, calculadas pela razão entre os casos do agravo e a população do período estudado, multiplicada por 100 mil. Como população, considerou-se a média das estimativas de mulheres referentes ao período e local do estudo, obtidas a partir das projeções populacionais do Brasil e das UF, por sexo e idade simples, do Instituto Brasileiro de Geografia e Estatística. Não foram calculadas taxas para raça/cor da pele, escolaridade, situação conjugal e gestante, pois não são disponibilizas projeções dessas características.

Obteve-se a variação percentual das taxas de notificação de violência no país com intervalo de confiança de 95% (IC95%). O coeficiente de variação percentual foi obtido fazendo-se a subtração entre o último ano e o primeiro, dividindo-se em seguida o resultado pelo valor do primeiro ano, multiplicado por 100; e o intervalo de confiança, a partir da multiplicação entre o erro-padrão e o t crítico de 1,96. 

### Controle de viés

O processamento se deu com a exclusão de todos os dados fora dos critérios de inclusão. Essa etapa foi realizada minuciosamente, analisando-se cada variável com filtros para minimizar dados faltantes e erros de preenchimento que pudessem levar à exclusão inadequada. Foram considerados dados faltantes aqueles cujo preenchimento do campo foi “ignorado” ou deixado em branco. Esse n e o percentual amostral foram descritos nos resultados, permitindo visualizar sua representatividade.

### Métodos estatísticos

Foi estimado o excesso de risco (*excess risk*) de ocorrência do agravo nas UF. Trata-se de uma comparação entre o número de casos com o número esperado a partir da consideração de um risco de referência, o qual é a agregação de todos os casos do país. O excesso de risco é obtido da comparação das taxas a um padrão nacional ou regional. Para o número esperado, foi realizada a razão entre a soma total dos casos e a soma das populações consideradas no estudo (19). Sendo assim, o cálculo para estimar o excesso de risco de cada UF foi a razão entre o número de eventos observado e o número esperado. Excesso de risco <1 representou UF com risco menor que a média nacional, e excesso de risco >1 representou UF com risco maior que a média do país (19).

Foi utilizado o Excel para tabulação, o software R 4.2.0 para o processamento e análises descritivas, o GeoDa 1.20.0.0 para os cálculos das taxas e excesso de risco, e o QGIS 3.16.16 para confecção dos mapas.

## Resultados

Foram analisadas 495.820 notificações de violência doméstica contra mulheres na faixa etária de 12 a 59 anos, no Brasil, de 2015 a 2020. A variação percentual das taxas de 2015 a 2020 foi de 29,4% (88,39 por 100 mil mulheres em 2015; e 114,41 por 100 mil em 2020), sendo que em 2020 a variação foi negativa em relação a 2019: -16,6% (137,12 notificações por 100 mil em 2019). Nesse mesmo período, as maiores taxas de violência foram na faixa etária de 12 a 14 anos (110,39 casos por 100 mil em 2015; e 165,63 por 100 mil em 2020), seguida pela de 20 a 29 anos (108,81 por 100 mil em 2015; e 144,34 por 100 mil em 2020) (Figura 1). 

**Figura 1:** Taxas de notificação de violência doméstica contra mulheres (por 100 mil), segundo faixa etária. Brasil, 2015-2020 (n=495.820)

Mulheres brancas representam 40% das notificações, e pardas 41,1%, sendo 36.338 dados faltantes (7,33%). Em todas as regiões do país, foi maior a proporção de vítimas com escolaridade mais alta (>8 anos), 43,8%. Porém, é alto o número de notificações de pessoas com escolaridade ignorada ou não preenchida (29,5%). Na situação conjugal, foram observadas maiores proporções de notificação de mulheres solteiras (47,2%), sendo que 58.887 eram dados faltantes (11,9%). Além disso, 76,5% das vítimas não estavam gestantes no momento da notificação, sendo 72.097 (14,5%) dados faltantes (Tabela 1). 

**Tabela 1:** Números absolutos (n), proporções (%), taxas de notificações (Tx) e variação percentual (Var %), bem como seus intervalos de confiança de 95% (IC95%), da violência doméstica contra a mulher, segundo características da vítima e região. Brasil, 2015-2020 (n=495.820)

Variáveis	Norte (n=34.219)		Nordeste (n=69.367)		Sudeste (n=266.601)		Sul (n=96.166)		Centro-Oeste (n=29.407)		Brasil(n=495.820)		Var (%)^a^
	n (%)	Tx.	n (%)	Tx.	n (%)	Tx.	n (%)	Tx.	n (%)	Tx.	n (%)	Tx.	(IC95%)
**Faixa etária** (anos)													
12-14	7.525 (21,9)	1.498,9	8.037 (11,6)	579,0	1.4274 (5,3)	827,0	9.749 (10,1)	1679,8	3.588 (12,2)	1.013,8	43.181 (8,7)	948,9	50,04 (16,61; 83,47)
15-19	5.516 (16,1)	642,3	8.537 (12,3)	346,9	32.961 (12,4)	1.047,4	13.900 (14,4)	1296,0	4.239 (14,4)	681,5	65.162 (13,1)	798,4	23,64 (-6,19; 53,47)
20-29	8.637 (25,2)	523,6	19.218 (27,1)	387,9	82.829 (31,1)	1.211,2	25.354 (26,4)	1079,8	8.259 (28,1)	614,0	144.311 (29,1)	842,2	32,65 (5,80; 59,49)
30-39	7.504 (21,9)	510,7	18.808 (27,1)	397,7	74.715 (28,0)	1.031,1	23.601 (24,5)	1013,1	7.305 (24,8)	531,9	131.952 (26,6)	769,5	25,67 (1,35; 49,98)
40-49	3.668 (10,7)	338,1	10.343 (14,9)	271,5	42.544 (15,9)	673,1	15.248 (15,9)	733,9	4.197 (14,8)	364,2	76.005 (15,3)	526,2	36,13 (10,53; 61,73)
50-59	1.369 (4,0)	188,3	4.424 (6,4)	149,7	19.278 (7,2)	351,5	8.314 (8,6)	433,6	1.819 (6,2)	208,0	35.209 (7,1)	294,4	32,55 (9,48; 55,63)
**Raça/cor da pele**													
Branca	3.976 (11,6)	-	9.140 (13,2)	-	108.684 (40,8)	-	69.277 (72,0)	-	7.220 (24,5)	-	198.326 (40,0)	-	-
Preta	2.339 (6,8)	-	7.555 (10,9)	-	30.854 (11,6)	-	6.106 (6,3)	-	2.476 (8,4)	-	49.332 (9,9)	-	-
Parda	25.442 (74,3)	-	45.957 (66,2)	-	100.995 (37,9)	-	15.974 (16,6)	-	15.199 (51,7)	-	203.589 (41,1)	-	-
Amarela e indígena	1.673 (4,9)	-	1.062 (1,5)	-	2.613 (0,9)	-	999 (1,0)	-	1.885 (6,4)	-	8.235 (1,67)	-	-
**Escolaridade** (anos)													
<8	12.734 (37,2)	-	21.224 (30,6)	-	59.402 (22,3)	-	30.111 (31,3)	-	8.923 (30,3)	-	132.409 (26,7)	-	-
≥8	15.411 (45,0)	-	25.671 (37,0)	-	119.666 (44,9)	-	45.787 (47,6)	-	10.609 (36,1)	-	217.167 (43,8)	-	-
**Situação Conjugal**													
Casada	12.610 (36,8)	-	26.603 (38,3)	-	110.428 (41,4)	-	40.924 (42,6)	-	12.433 (42,3)	-	203.016 (40,9)	-	-
Solteira	18.564 (54,2)	-	34.347 (49,5)	-	120.301 (45,1)	-	47.752 (49,7)	-	12.917 (43,9)	-	23.3917 (47,2)	-	-
Gestante													
Sim	6.666 (19,5)	-	7.920 (11,4)	-	17.434 (6,5)	-	9.119 (9,5)	-	3.394 (11,5)	-	44.539 (8,9)	-	-
Não	23.766 (69,4)	-	50.961 (73,5)	-	203.386 (76,3)	-	79.371 (82,5)	-	21.659 (73,6)	-	379.184 (76,5)	-	-

^a^VAR = Variação percentual de 2015 a 2020

Um total de 54,9% das vítimas estava sofrendo violência de repetição (80.766 notificações não categorizadas -16,3%). Entre os tipos de violência, a física obteve maior taxa (534,9 por 100 mil), seguida pela psicológica (295,2 por 100 mil). O meio de agressão mais observado foi o uso de força corporal/espancamento (71,2%) e ameaças (28,2%). A taxa de notificação de violência sexual foi 70,7 por 100 mil (10,5%), sendo o estupro o tipo mais evidente (51,1 por 100 mil), tendo destaque a região Norte, com taxa de violência sexual de 156,9 por 100 mil (Tabela 2).

**Tabela 2:** Números absolutos (n), proporções (%) e taxas de notificações (Tx) da violência doméstica contra a mulher, segundo características da violência e região. Brasil, 2015-2020 (n=495.820)

Variáveis	Norte (n=34.219)		Nordeste (n=69.367)		Sudeste (n=266.601)		Sul (n=96.166)		Centro-Oeste (n=29.407)		Brasil (n=495.820)	
	n (%)	Tx	n (%)	Tx.	n (%)	Tx.	n (%)	Tx.	n (%)	Tx.	n (%)	Tx.
**Violência de repetição**												
Sim	20.690 (60,5)	328,9	38.955 (56,2)	191,9	140.984 (52,9)	458,3	56.396 (58,6)	546,2	15.395 (52,3)	269,1	272.447 (54,9)	371,2
Não	9.100 (26,6)	144,6	18.490 (26,7)	91,1	78.054 (29,3)	253,7	28.413 (29,5)	275,2	8.524 (28,9)	149,0	142.607 (28,8)	194,3
Tipo de violência ^a^												
Física	20.053 (58,6)	318,7	50.759 (73,2)	250,1	230.279 (86,4)	748,6	68.409 (71,1)	662,5	23.053 (78,4)	402,9	392.602 (79,2)	534,9
Psicológica	16.198 (47,3)	257,5	36.518 (52,6)	179,9	108.849 (40,8)	353,8	44.405 (46,2)	430,0	10.662 (36,3)	186,4	216.660 (43,7)	295,2
Sexual	9.875 (28,9)	156,9	11.713 (16,9)	57,7	16.554 (6,2)	53,8	9.544 (9,9)	92,4	4.172 (14,2)	72,9	51.869 (10,5)	70,7
Outros tipos de violência	3.060 (8,9)	48,6	11.858 (17,1)	58,4	23.052 (8,6)	74,9	15.095 (15,7)	146,2	3.748 (12,7)	65,5	56.826 (11,5)	77,4
Meio de Agressão ^a^												
Força corporal / espancamento	17.119 (50,0)	272,1	41.926 (60,4)	206,6	210.461 (78,9)	684,2	63.313 (65,8)	613,1	20.067 (68,2)	350,8	352.927 (71,2)	480,8
Enforcamento	1.650 (4,8)	26,2	4.482 (6,5)	22,1	22.243 (8,3)	72,3	5.150 (5,4)	49,9	2.393 (8,1)	41,8	35.919 (7,2)	48,9
Objeto contundente	1.772 (5,2)	28,2	4.331 (6,2)	21,3	14.142 (5,3)	45,9	4.396 (4,6)	42,6	1.919 (6,5)	33,5	26.563 (5,4)	36,2
Objeto perfuro-cortante	2.735 (7,9)	43,5	7.276 (10,5)	35,8	17.017 (6,4)	55,3	6.194 (6,4)	59,9	3.056 (10,4)	53,4	36.287 (7,3)	49,4
Ameaça	10.996 (32,1)	174,8	23.576 (33,4)	116,1	63.899 (23,9)	207,7	34.496 (35,9)	334,1	6.746 (22,9)	117,9	139.729 (28,2)	190,4
Outros meios	3.842 (11,5)	61,2	8.811 (13,4)	43,4	19.199 (7,5)	62,4	10.449 (10,9)	101,2	3.300 (11,6)	57,7	45.611 (9,2)	62,1
**Violência sexual**												
Assédio	2.272 (6,6)	36,1	2.682 (3,9)	13,2	4.928 (1,8)	16,0	2.740 (2,8)	26,5	869 (2,9)	15,2	13.492 (2,7)	18,4
Estupro	7.040 (20,6)	111,9	8.249 (11,9)	40,6	11.749 (4,4)	38,2	7.156 (7,4)	69,3	3.281 (11,2)	57,3	37.484 (7,6)	51,1
Outros tipos	920 (2,7)	14,6	1.492 (2,1)	7,3	1.606 (0,6)	5,2	868 (0,9)	8,4	391 (1,3)	6,8	5.279 (1,1)	7,2

^a^As vítimas podem ter sofrido mais de um tipo de violência e mais de um tipo de agressão

Observam-se maiores taxas de cônjuges como provocadores da violência, seguidos por ex-cônjuges e namorados (291; 118,4 e 62,6 casos por 100 mil, respectivamente). Quanto ao encaminhamento das vítimas, quando realizado, a maior taxa foi para serviços não especializados, como delegacias, redes de saúde, entre outros (673,5 por 100 mil). A principal motivação da violência foi “outras motivações”, porém em 40% dos dados o campo foi ignorado ou não preenchido (Tabela 3).

**Tabela 3:** Números absolutos (n), proporções (%) e taxas de notificações (Tx) da violência doméstica contra a mulher, segundo características do agressor, encaminhamento, motivação e região. Brasil, 2015-2020 (n=495.820)

Variáveis	Norte (n=34.219)		Nordeste (n=69.367)		Sudeste (n=266.601)		Sul (n=96.166)		Centro-Oeste (n=29.407)		Brasil (n=495.820)	
	n (%)	Tx	n (%)	Tx.	n (%)	Tx.	n (%)	Tx.	n (%)	Tx.	n (%)	Tx.
Agressor^a^												
Pai	1.470 (4,3)	23,4	3.270 (4,7)	16,1	14.032 (5,3)	45,6	8.111 (8,4)	78,5	1.904 (6,5)	33,3	28.792 (5,8)	39,2
Mãe	898 (2,6)	14,3	2.707 (3,9)	13,3	12.833 (4,8)	41,7	9.122 (9,5)	88,3	1.822 (6,2)	31,8	27.387 (5,5)	37,3
Padrasto	1.635 (4,8)	25,9	1.754 (2,5)	8,6	6.346 (2,4)	20,6	3.090 (3,2)	29,9	1.075 (3,7)	18,8	13.902 (2,8)	18,9
Madrasta	82 (0,2)	1,3	153 (0,2)	0,7	884 (0,3)	2,9	292 (0,3)	2,8	90 (0,3)	1,6	1.501 (0,3)	2,0
Cônjuge	12.748 (37,2)	202,6	29.557 (42,6)	145,6	117.386 (44,0)	381,6	40.475 (42,1)	391,9	13.400 (45,6)	234,2	213.589 (43,1)	291,0
Ex-cônjuge	6.754 (19,7)	107,4	13.062 (18,8)	64,3	46.128 (17,3)	149,9	16.601 (17,3)	160,8	4.338 (14,7)	75,8	86.892 (17,5)	118,4
Namorado	5.449 (15,9)	86,6	7.318 (10,5)	36,0	22.962 (8,6)	74,6	7.268 (7,6)	70,4	2.945 (10,0)	51,5	45.953 (9,3)	62,6
Ex-namorado	1.627 (4,7)	25,9	3.500 (5,0)	17,2	15.229 (5,7)	49,5	4.188 (4,3)	40,6	1.078 (3,7)	18,8	25.626 (5,2)	34,9
Filho	526 (1,5)	8,4	1.725 (2,5)	8,5	10.625 (3,9)	34,5	3.839 (3,9)	37,2	1.110 (3,8)	19,4	17.825 (3,6)	24,3
Irmão	1.463 (4,3)	23,2	3.546 (5,5)	17,5	16.665 (6,2)	54,2	4.303 (4,5)	41,7	1.487 (5,1)	25,9	27.467 (5,5)	37,4
Outro familiar	2.524 (7,4)	40,1	6.129 (8,8)	30,2	18.287 (6,9)	59,4	7.165 (7,4)	69,4	2.171 (7,4)	37,9	36.279 (7,3)	49,4
Sexo do agressora												
Masculino	31.745 (92,8)	504,6	62.427 (90,0)	307,6	230.833 (85,6)	750,4	80.976 (84,2)	784,2	25.467 (86,6)	445,2	431.500 (87,0)	587,9
Feminino	1.650 (4,8)	26,2	4.031 (5,8)	19,9	22.261 (8,3)	72,4	8.067 (8,4)	78,1	2.274 (7,7)	39,7	38.286 (7,7)	52,2
Ambos	536 (1,6)	8,5	1.728 (2,5)	8,5	9.364 (3,5)	30,4	6.648 (6,9)	64,4	1.337 (4,5)	23,4	19.616 (3,9)	26,7
**Suspeita de uso de álcool**												
Sim	11.840 (34,6)	188,2	26.140 (37,7)	128,8	96.755 (36,3)	314,5	36.007 (37,4)	348,7	13.211 (44,9)	230,9	183.975 (37,1)	250,7
Não	15.849 (46,3)	251,9	27.041 (38,9)	133,2	103.293 (38,7)	335,8	38.869 (40,4)	376,4	9.539 (32,4)	166,7	194.622 (39,2)	265,2
Encaminhamento^b^												
Serviço especializado	14.154 (41,4)	224,9	27.862 (40,2)	137,3	85.962 (32,2)	279,4	32.647 (33,9)	316,2	8.955 (30,4)	156,5	169.602 (34,2)	231,1
Outros serviços	29.359 (85,8)	466,7	55.386 (79,8)	272,9	256.837 (96,3)	834,9	121.866 (126,7)	1180,2	30.837 (104,9)	539,0	494.339 (99,7)	673,5
**Motivação da violência**												
Sexismo	7.721 (22,6)	122,7	20.227 (29,2)	99,6	53.204 (19,9)	172,9	22.746 (23,6)	220,3	5.206 (17,7)	91,0	109.113 (22,0)	148,7
Conflito Geracional	2.909 (8,5)	46,2	5.574 (8,0)	27,5	37.239 (13,9)	121,0	9.206 (9,6)	89,2	2.663 (9,1)	46,5	57.599 (11,6)	78,5
Outras motivações	7.630 (22,3)	121,3	15.868 (22,9)	78,2	67.002 (25,1)	217,8	32.002 (33,3)	309,9	8.094 (27,5)	141,5	130.617 (26,3)	177,9

^a^As vítimas podem ter mais de um agressor; b As vítimas podem ter sido encaminhadas para mais de um serviço.

No mapa de distribuição das taxas de notificação de violência (Figura 2), os estados com maiores valores foram Acre (1.304,45 por 100 mil), Tocantins (971,71 por 100 mil), Minas Gerais (1.020,53 por 100 mil), Mato Grosso do Sul (1.271,74 por 100 mil), Paraná (1.122,67 por 100 mil) e Rio Grande do Sul (969,12 por 100 mil). Os estados de Rondônia, Maranhão, Piauí, Rio Grande do Norte, Paraíba e Sergipe apresentaram as menores taxas do país.

**Figura 2:** Taxas de notificação de violência doméstica contra mulheres (por 100 mil) nas unidades federativas. Brasil, 2015-2020 (n=495.820)

Quanto ao excesso de risco, os estados onde o risco do agravo foi maior que a média de todo o país foram: Acre (1,93), Mato Grosso do Sul (1,88), Paraná (1,66), Minas Gerais (1,51), Tocantins (1,43), Rio Grande do Sul (1,43), Rio de Janeiro (1,22), São Paulo (1,21) e Espírito Santo (1,17).

## Discussão

O estudo mostrou uma variação percentual positiva das taxas de notificação de violência doméstica contra mulheres no Brasil, ao longo do período, e decréscimo em 2020. O agravo atinge mulheres de todas as raças/cores e níveis de escolaridade, e encontra-se amplamente distribuído em todo o Brasil, porém as maiores proporções são observadas em mulheres brancas, pardas e com maior escolaridade, e alguns estados apresentam taxas de notificação maiores que a média do país.

A redução nas taxas de violência é evidente em 2020, início da pandemia de covid-19. A partir do isolamento social adotado para conter o avanço do coronavírus (Sars-CoV-2), a violência doméstica contra mulheres foi agravada em diversos países, visto que ocorre, em sua grande maioria, em situações de afetividade ou coabitação, e a medida tornou a convivência no domicílio constante, possibilitando aumento de discussões e tensões (20). Além disso, o medo de contaminação e mudanças nos serviços de atendimento às mulheres também podem ter provocado o decréscimo na procura de instituições para denúncias (20). Houve uma queda de 7,4% nos registros de lesão corporal por violência doméstica, mas, ainda assim, 230.160 mulheres realizaram denúncias (aproximadamente 630 por dia) (21); porém, isso não necessariamente implica a diminuição dos casos, visto que as mudanças impostas pela quarentena aumentam a situação de vulnerabilidade (22). Aliás, os dados de ligações para o número 190 (emergência policial) referentes à violência doméstica indicam crescimento de 16,3% em relação a 2019 (23), e os registros de feminicídio aumentaram 22,2% em março e abril de 2020 (21). Os números de notificações dependem de inúmeros fatores, como o desenvolvimento dos serviços de saúde, ou até mesmo a procura das vítimas. Segundo a Pesquisa Nacional de Saúde de 2019, 19,4% das mulheres relataram ter sofrido algum tipo de violência; entretanto, apenas 16,9% procuraram atendimento em saúde (24).

As maiores taxas de notificação são em mulheres mais jovens, de 12 a 14 anos e 20 a 29 anos, indicando a necessidade de um maior aprofundamento para que se entendam as causas e fatores associados nessas faixas etárias. Registros realizados por escolas públicas são relevantes, constituindo um setor importante para os encaminhamentos e notificações nas faixas de idade escolar (25). Neste estudo, dados de vítimas adolescentes foram altamente representativos; em relação a essa fase da vida, as escolas podem possuir um papel importante nas notificações de violência a partir da identificação das vítimas e encaminhamento para os serviços responsáveis.

Uma pesquisa realizada com dados de notificação de 2008 a 2017, no município do Rio de Janeiro, apontou que as faixas etárias mais acometidas por violência doméstica foram de 20 a 29 anos (29,1%) e 30 a 39 anos (22%) (26), corroborando os presentes achados, em que os maiores percentuais foram observados nessas faixas etárias. Um estudo realizado no Zimbábue, com dados de 2005, 2006, 2010, 2011 e 2015, identificou que mulheres de 15 a 19 anos possuem maior chance de sofrer violência doméstica em comparação a mulheres mais velhas, mostrando a vulnerabilidade do grupo frente ao agravo, o qual pode não compreender as complexidades dessa violência dentro de relacionamentos (27).

Também é observada grande proximidade entre as proporções de notificação de violência contra mulheres pardas e brancas. Uma pesquisa realizada no Brasil, com dados de notificação de 2009 a 2014, apontou que a raça/cor da pele parda obteve maiores taxas nas regiões Norte, Nordeste e Centro-Oeste, enquanto a raça/cor da pele branca foi mais representativa nas regiões Sul e Sudeste, corroborando amplamente os nossos achados (7). Entretanto, no Brasil predominam pessoas autodeclaradas pardas, não sendo possível afirmar com proporções as vítimas mais atingidas (28).

Observamos maiores proporções do agravo em mulheres com maior escolaridade. Embora a baixa escolaridade seja representativamente apresentada por estudos com maiores prevalências e como fator de risco, algumas pesquisas encontraram maiores taxas de violência em vítimas com níveis mais altos de escolaridade (26,29). Ainda assim, mulheres de todos os níveis são atingidas (29). 

As altas proporções de violência em mulheres com maior escolaridade também podem ser reflexo do acesso à informação e desenvolvimento de instituições de segurança, educação e saúde. Por meio de campanhas publicitárias, essas instituições disseminam informações relevantes sobre o agravo, as medidas protetivas e os direitos das mulheres, estimulando, assim, a autonomia para que elas interrompam o ciclo de violência (30).

Vale ressaltar, ainda, que a maior parte das mulheres não estava sofrendo violência doméstica pela primeira vez, caracterizando violência de repetição, o que também foi observado por outras pesquisas (26,31). Um estudo realizado em 2020, em Salvador, Bahia, com mulheres com representação judicial por violência conjugal, evidenciou que as agressões sofridas se intensificam ao longo do relacionamento, até que se tornam mais graves e menos veladas (32). A permanência frente à repetição muitas vezes constitui também uma estratégia de proteção, visando evitar uma violência mais grave, como o feminicídio, onde uma resistência da mulher pode ter consequências se o agressor tentar impedir a mesma de deixá-lo (33). Apoio externo é identificado como um dos fatores importantes para que as mulheres saiam, da situação de violência doméstica em que se encontra, juntamente com o medo dos impactos negativos da violência na saúde física e mental e a necessidade de proteção das crianças (34).

A violência física é a principal tipologia encontrada neste estudo, indo ao encontro dos resultados apontados em diversas pesquisas (26,29,31). Ela possui uma importante magnitude, especialmente pelo risco de causar lesões evidentes (29). A violência psicológica também possui altas taxas, divergindo dos resultados de Silva e Oliveira (29), com dados de notificação de 2009 a 2012 no Distrito Federal, em que a violência sexual aparece logo após a violência física, seguida pela psicológica. Porém, deve-se levar em consideração que, normalmente, a violência psicológica é uma das primeiras manifestações, mas muitas vezes é ignorada (32).

O meio de agressão mais utilizado foi a força corporal/espancamento, seguido pela ameaça, indo ao encontro de um estudo realizado no município do Rio de Janeiro, com notificações de 2008 a 2017 (26), e contra os achados de outra pesquisa em nível nacional, de 2009 a 2014, em que todas as regiões obtiveram ameaça com maior número de registros (7). 

A maior parte das vítimas foi encaminhada para serviços não especializados. A rede de atendimento para mulheres em situação de violência possui várias portas de entrada, as quais realizam posterior encaminhamento, porém muitas vezes esse encaminhamento não ocorre de forma adequada, ocasionando lacunas na integração de serviços (35).

O estudo permite corroborar o fato de que a violência doméstica ultrapassa barreiras sociodemográficas, atingindo todas as mulheres. Sendo assim, deve-se considerar o contexto em que as vítimas estão inseridas, e não apenas um fator isolado (36,37).

Neste estudo, o agravo se encontra distribuído em todo o país. Entretanto, foi possível identificar os estados com maior excesso de risco. Estados com altas taxas também apresentaram excesso de risco >1. 

As maiores taxas de violência foram observadas no Acre, Tocantins, Minas Gerais, Mato Grosso do Sul, Paraná e Rio Grande do Sul, se assemelhando aos achados da distribuição espacial realizada por outro estudo (38). Outra pesquisa apontou o estado do Mato Grosso do Sul como detentor do maior coeficiente de notificação de violência contra as mulheres (7), o que se assemelha ao presente estudo, visto que esse estado ocupou a segunda posição de maior taxa e de maior excesso de risco, atrás apenas do Acre.

Entre as limitações do estudo, o uso de banco de dados secundários é uma das principais, onde variáveis apresentam proporções significativas de dados preenchidos como “ignorado”, “outros”, “não se aplica”, ou não preenchidos. Contudo, o uso desses registros não invalida os achados, visto que todas as variáveis obtiveram uma completude maior que 70%, exceto pela variável motivação, com 59,97% de completude, requerendo cautela na interpretação. Também não obtivemos as características da violência considerando todas as mulheres do país, pela exclusão de crianças e idosas, visto que são necessários estudos voltados para esses grupos, devido às violências nessas faixas possuírem tratativas diferenciadas, outras motivações e formas de agressão. Para minimizar a perda de dados, o processamento foi minucioso, com a verificação das variáveis separadamente, visando suprir a ausência de informações.

Não foi possível calcular as taxas por raça/cor da pele, escolaridade, situação conjugal e gestação, devido à inexistência de estimativas populacionais. Ainda, a raça/cor da pele das notificações pode prover da observação do profissional notificador, ocasionando também um possível viés, e muitos trabalhadores podem não considerar a importância de notificar eventos de violência e pacientes podem não a reconhecer como responsabilidade dos sistemas de saúde, ignorando a necessidade de denúncia, fatos que podem contribuir para a variação das notificações a partir do contexto de cada UF.

Contudo, a violência doméstica contra mulheres no Brasil apresentou importantes variações entre 2015 e 2020, com aumento contínuo das taxas em todos os anos, exceto em 2020, ano em que houve uma queda representativa. Mulheres mais jovens, especialmente entre 12 e 14 anos, apresentaram maiores taxas de notificação, enquanto mulheres brancas, pardas e com maior escolaridade apresentaram maior proporção. Além disso, a análise espacial revelou estados que apresentaram uma taxa do agravo maior que a média nacional, ocasionando um maior excesso de risco. Esses achados destacam as desigualdades na distribuição do agravo entre grupos populacionais e regiões do país, reforçando a necessidade de abordagens direcionadas, além de análises aprofundadas e ação coordenada de serviços e profissionais para enfrentamento.

Dessa forma, conclui-se que a violência doméstica contra mulheres é um fenômeno complexo e heterogêneo, sendo este estudo uma importante fonte de dados, com resultados relevantes sobre o cenário do agravo, contribuindo para elaboração de estratégias de enfrentamento. Além disso, provê subsídios para discussões, visando especialmente ao conhecimento do perfil das vítimas, à qualificação de políticas públicas já existentes e à adequação profissional e institucional para atendimento e combate efetivos.

## Data Availability

O banco de dados utilizado para o estudo está disponível de forma pública pelo link https://datasus.saude.gov.br/transferencia-de-arquivos/, através dos filtros: fonte – SINAN, modalidade – dados, tipo de arquivo – viol (violência doméstica, sexual e/ou outras violências, ano – 2015 a 2020, UF – BR. Os conteúdos utilizados para o estudo também se encontram disponíveis. Segue títulos e respectivas URLs, números de acesso ou DOIs dos arquivos dos conteúdos subjacentes ao texto do artigo: Dados do SINAN: https://datasus.saude.gov.br/wp-content/zipupload/Arq_162698079/arquivo.zip Script R para processamento: https://github.com/laysaz/artigo1_disser_lays Projeto GQis para mapeamento: https://github.com/laysaz/artigo1_disser_lays
